# Zonal origin of prostate cancer: comparison of long-term outcomes after radical prostatectomy

**DOI:** 10.1007/s11255-023-03637-7

**Published:** 2023-05-31

**Authors:** Laura Luttrell, Jian Li, Ronald J. Cohen

**Affiliations:** 1 Medical School, University of Western Australia, Perth, WA Australia; 2 Uropath Pty Ltd, 2/47 Oxford Close, West Leederville, Perth, WA 6007 Australia

**Keywords:** Adjuvant therapy, Biochemical recurrence, Prostate cancer, Prostatectomy, Zonal origin

## Abstract

**Purpose:**

To assess the impact of zonal origin on positive bladder neck (BN) margins and prostate-specific antigen (PSA) failure or early adjuvant therapy in the patients with complete long-term follow-up after radical prostatectomy (RP).

**Methods:**

A set of 4512 men were identified who underwent RP in Western Australia (WA) from March 2000 to December 2016 and had complete long-term follow-up. The *t*-test, Pearson chi-square test and Kaplan–Meier method with the log-rank test were used to evaluate differences between the transition zone (TZ) and peripheral/central zone (PZ/CZ) cancer. Univariate and multivariable Cox proportional hazard regression models were applied to assess parameters on PSA failure and early treatment.

**Results:**

The positive BN margin rate for TZ cancer fell significantly over the study period. However, BN margin rates increased for PZ/CZ cancer over the time. Data of 4512 patients with median follow-up of 9.1 years confirmed that the high-risk TZ tumours with negative margins had a significant lower rate of PSA failure or early treatment compared to those high-risk PZ/CZ tumours with negative margins.

**Conclusion:**

Prostate cancer zonal origin significantly impacts long-term biochemical outcomes in high-risk and margin-negative patients. BN invasion with margin involvement is more frequent in the TZ cancer and can be reduced by pre-operative identification of cancer zonal origin and adjustment of surgical procedures.

## Introduction

Index prostatic TZ adenocarcinomas have been previously reported in up to 25% of RP specimens [1, 2]. It has been observed that tumours originating from this zone are more likely to be of lower grade and larger volume and associated with higher levels of serum PSA at the time of RP when compared to PZ and CZ tumours [3, 4]. Their egress from the gland is often via the BN rather than via the posterolateral nerve bundles as seen in PZ carcinomas or via the seminal vesicles as seen in CZ tumours [4–6]. Due to their lower histologic grade, TZ tumours were previously deemed to have a more favourable prognosis [3, 4]. Positive surgical margins in TZ tumours were mostly seen at the BN, anteriorly or at the distal urethra [5].

The prostate gland has been shown to have an apocrine-type secretion where epithelial cells release their contents into gland lumina via a mechanism of decapitation secretion [7, 8]. Protease enzymes together with spermine are packaged into secretory organelles called prostate secretory granules. This mechanism of secretion is diminished in high-grade prostatic intraepithelial neoplasia and in PZ and CZ carcinoma [9]. By contrast, the mechanism is well preserved, possibly over regulated, in TZ tumours thereby enabling their histological identification on pre-operative needle core biopsies preserved in glutaraldehyde-based tissue fixatives [10]. A nomogram has been developed utilising these TZ histologic features (granules content, clear cells, luminal secretions) on needle core biopsy to predict the risk of subsequent BN margin positivity at RP [11]. Using these histologic features, all prostate biopsies were preserved in glutaraldehyde-based fixative and all reports from January 2017 included information as to the likely presence of TZ adenocarcinoma. This alerted the attending surgeon to the presence of TZ tumours and allowed pre-operative planning of a wider BN resection in an attempt to reduce BN margin positivity.

It has been recognised that after RP, many patients may receive adjuvant therapy before PSA values reach a level indicative of therapeutic failure (0.2 ng/mL). In this series 14.7% patients who had positive margins at RP received adjuvant therapy (hormone therapy and radiotherapy) without PSA elevation above 0.2 ng/mL. Thus, using only PSA failure as a criterion to assess differences between TZ and PZ/CZ cancer without consideration of early treatment cases may be inaccurate [3, 5, 12, 13]. In this study, we took both PSA failure (PSA ≥ 0.2 ng/mL) and initiation of early adjuvant treatment as criteria to evaluate impact of the zonal origin in patients with low- and high-grade disease.

## Patients and methods

### Patient selection

The West Australian RP database contains 11,901 cases treated by 29 urological surgeons since 1998. Of these 29 surgeons, 11 surgeons have complete follow-up for all cases representing 6933 men. From this subset of 6933 men with complete follow-up history, 2421 were identified between 2017 and 2020, and 4512 patients who underwent RP between 2000 and 2016 had complete long-term follow-up for analysis. Clinicopathological data were retrieved from the datasets including pre-operative PSA level, core biopsy and RP specimen tumour zone origin, tumour volume, positive surgical margin (PSM), intraductal carcinoma (IDCP), extraprostatic spread (EPS), seminal vesicle invasion (SVI), Gleason sum (GS), post-operative PSA and subsequent further therapy. Biochemical recurrence (BCR) was defined as confirmed PSA value of 0.2 ng/mL or greater.

The 4512 patients with the long-term follow-up were then divided into two groups: high risk vs low risk. The high-risk group was defined as GS 4 + 3, 8 and 9 or greater, i.e. International Society of Urological Pathology (ISUP) groups 3, 4 and 5. The low-risk group was identified as GS 6 or less and 3 + 4, i.e. ISUP groups 1 and 2.

### Biopsy and radical prostatectomy specimens

The index tumour zonal origin was reported in all RP pathological reports. The pre-operative biopsy samples from 2015 onwards were assessed for the presence of TZ adenocarcinoma after fixation in a glutaraldehyde tissue fixative (Solufix) [7]. Biopsy and RP specimens were examined by two uropathologists for standard pathological interpretation and specific zonal characters. The risk of BN invasion and tumour extension to BN margin was assessed by our pre-operative published nomogram utilising biopsy determined pathological features [10, 11].

### Statistical analysis

The clinical and pathological characteristics between TZ and PZ/CZ cancers were compared by the *t*-test and the Pearson *χ*^2^ test. The Kaplan–Meier method with the log-rank test was used to evaluate differences in BCR/early treatment between different selected groups. Univariate and multivariable Cox proportional hazards regression models were applied to assess the impact of potential predictors on BCR/early treatment after RP. All statistical analyses were performed with IBM SPSS statistics, version 20 and all tests were two sided with a significance level of 0.05.

## Results

### Incidence of a PSM in RP specimens (whole database of 11901)

Assessment of 11,901 patients from the RP database confirms a PSM rate that has significantly fallen over the years. This is most evident in TZ cancer, where the PSM rate has decreased from 27.26% between the years 1998–2016 to 18.14% between 2017 and 2020 (*p* < 0.001). During the same time period, the PSM rate of PZ/CZ tumours fell from 19.10 to 16.00% (*p* = 0.001). The percentage of positive BN margins in all margin-positive TZ cancer fell over the same time period from 41.43 to 30.97% (*p* = 0.042). This contrasts with PZ/CZ carcinomas where BN margins as a percentage of all positive margins rose minimally over the same time period from 11.74 to 13.78% (*p* = 0.293).

During the same period, the averages of the GS increased significantly from 6.67 to 7.16 in TZ cancer (*p* < 0.001) and from 7.10 to 7.47 in PZ/CZ cancer (*p* < 0.001) but there was no statistical difference in tumour volume for TZ (4.66 cc vs 4.95 cc, *p* = 0.244) or PZ/CZ cancer (3.48 cc vs 3.67 cc, *p* = 0.165).

### Subset of 4512 with complete long-term follow-up treated by 11 urological surgeons

#### Comparison of TZ and PZ/CZ PSA failure or early treatment

Long-term follow-up data of 4512 patients with complete information between 2000 and 2016 with a median follow-up of 9.1 years, confirmed no significant difference between low-risk, margin-negative TZ or CZ/PZ tumours with regards PSA failure or early treatment rates (*p* = 0.652). In assessing the data for margin-positive tumours, the failure or early treatment rates were also not statistically significant in comparing TZ and PZ/CZ tumours in both the low-risk and high-risk groups (*p* = 0.721 and 0.384). However, patients with TZ tumours that were high risk and margin negative were found to have a statistically significant lower rate of PSA failure or early treatment compared to patients with high-risk and margin-negative PZ/CZ tumours (*p* < 0.001).

#### Comparison of tumour volumes

In assessing tumour volume data for the high-risk patients with margin-negative and margin-positive disease, there was no difference in the volumes of TZ and PZ/CZ tumours, respectively (margin negative *p* = 0.180, margin positive *p* = 0.323). A statistically significant difference was seen, however, in the TZ and PZ/CZ tumour volumes in the low-risk groups for both margin-negative and -positive disease (both *p* < 0.001), respectively. TZ tumours were significantly larger in the low-risk groups regardless of margin status.

#### High risk tumours with negative margins

The clinicopathological characteristics (Table 1) of patients with TZ tumours were compared to those with PZ/CZ tumours in all cases of high-risk margin-negative disease. TZ tumours were significantly more likely to be observed in older aged men (mean age 64.8 vs 63.6, *p* = 0.035) and be associated with a higher pre-operative PSA level (mean PSA 9.21 vs 8.04 ng/mL, *p* = 0.020). They were significantly less likely to be associated with IDCP, EPS, SVI, BCR or early treatment (all *p* < 0.001). In this high-risk group with negative margins, there was no statistical difference in the number of men with GS ≥ 8 (*p* = 0.096) when comparing TZ to PZ/CZ tumours. We also compared the incidence of BCR and early adjuvant treatment between TZ and PZ/CZ tumours in GS = 4 + 3 as compared to GS ≥ 8. For the patients with GS = 4 + 3, 7.89% of 114 TZ cases had a BCR, while 14.55% of 976 PZ/CZ cases failed within 5 years (*p* = 0.061). On the other hand, 2.63% of TZ and 7.17% of PZ/CZ patients received the early adjuvant treatments (*p* = 0.074). However, if we combined the BCR and early treatment as criteria to evaluate impact of the zonal origin, TZ tumours had a significant lower incidence in BCR/early treatment compared to PZ/CZ tumours with GS = 4 + 3 (TZ 9.65% vs PZ/CZ 20.49%, *p* = 0.004). For the group of GS ≥ 8, 16.13% of 31 TZ cases and 37.57% of 378 PZ/CZ cases had a BCR (*p* = 0.019), and 9.68% of TZ and 15.34% of PZ/CZ patients received the early treatments (*p* = 0.599) within 5 years. There was a significant difference in BCR/early treatment between TZ and PZ/CZ tumours with GS ≥ 8 (TZ 22.58% vs PZ/CZ 47.88%, *p* = 0.008).

**Table 1:** Descriptive statistics for high-risk patients with negative margins: TZ vs PZ/CZ cancer

	TZ		PZ/CZ		
Variable	Value	Percent	Value	Percent	*p* value
Patients	145		1354		
Age, years					
Mean (median)	64.8 (65)		63.6 (64)		0.035
PSA, ng/mL					
Mean (median)	9.21 (7.6)		8.04 (6.7)		0.020
Tumour volume (cc)					
Mean (median)	4.79 (3.71)		4.22 (2.94)		0.180
ISUP group					
3 (GS 4 + 3)	114		976		0.096
4–5 (GS 8–10)	31	21.38	378	27.92	
IDCP	22	15.17	665	49.11	< 0.001
EPS	40	27.59	771	56.94	< 0.001
SVI	7	4.83	226	16.69	< 0.001
Lymph nodes					
Positive	0	0	84	6.20	
Negative	52	35.86	617	45.57	
Not done	93	64.14	653	48.23	
BCR or treated (≤ 5 years)	18	12.41	381	28.14	< 0.001

**Fig. 1:**
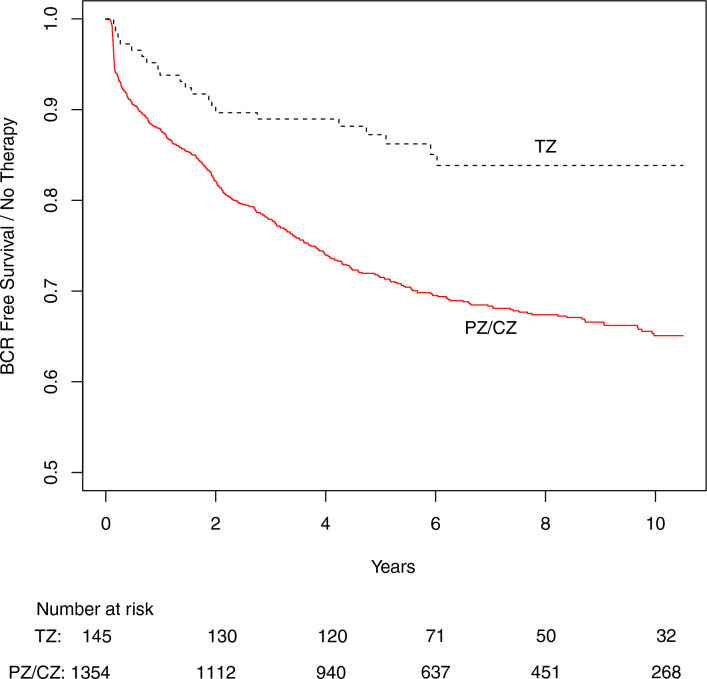
BCR-free survival/no therapy in 1499 patients with high-risk and margin-negative disease: TZ vs PZ/CZ cancer

Kaplan–Meier plot of BCR-free survival for patients with TZ versus PZ/CZ tumours in men identified as having high-risk (GS ≥ 4 + 3), margin-negative disease is shown in Fig. 1. Patients with TZ tumours had significantly longer BCR-free survival compared to patients with PZ/CZ tumours (*p* < 0.001). On both the univariate and multivariate analyses of the high-risk cancer with negative margins, the PSA level, GS, tumour volume, IDCP, EPS, SVI and PZ/CZ tumour origin were all associated with earlier BCR or early treatment (Table 2).

**Table 2:** Univariate and multivariable analyses in the high-risk group with negative margins

Variables	Univariate		Multivariable	
	Hazard ratio (95% CI)	*p* value	Hazard ratio (95% CI)	*p* value
PSA, ng/mL	1.06 (1.05–1.08)	< 0.001	1.05 (1.04–1.07)	< 0.001
Gleason sum	1.79 (1.61–1.99)	< 0.001	1.41 (1.26–1.58)	< 0.001
Tumour volume, mL	1.08 (1.07–1.09)	< 0.001	1.02 (1.01–1.04)	0.004
IDCP	2.93 (2.40–3.57)	< 0.001	1.97 (1.59–2.44)	< 0.001
EPS	3.07 (2.48–3.80)	< 0.001	1.53 (1.20–1.96)	0.001
SVI	4.30 (3.53–5.24)	< 0.001	2.02 (1.60–2.55)	< 0.001
TZ tumour origin	0.46 (0.30–0.71)	< 0.001	0.63 (0.40–0.99)	0.045

## Discussion

Previous studies have identified that TZ prostate cancers due to their anatomic location are more likely to have PSM at the BN in RP specimens than cancers originating from the PZ or CZ [12]. This study is the first to confirm the proposed value [11, 12] of identifying TZ carcinoma on pre-operative biopsy using a glutaraldehyde-based tissue fixative, and providing an individual risk profile of BN invasion allowing a wider resection of the BN at RP. Using this method [11], we have observed a significant reduction in the number of patients with TZ cancer having a positive BN margin at RP. An isolated positive BN margin has previously been associated with shorter time to BCR [12] and from this current analysis the importance of a negative BN margin is greatest in high-risk TZ cancer as compared to high-risk PZ/CZ cancer or tumours with low-risk morphology. In addition to BN margins, the incidence of all PSM (TZ/PZ/CZ tumours) have significantly fallen over the past 4 years, possibly highlighting other contributing factors such as improved early detection with MRI, better patient selection, increased numbers of core samples and increased compliance in PSA screening programmes in young men. However, in contrast to TZ cancer, the incidence of positive BN margins in PZ/CZ cancer has increased marginally during the same time period.

In this study, we conducted a comprehensive analysis to evaluate the impact of zonal origin on time to early treatment or PSA failure. Earlier studies were conflicting, with some providing evidence that tumour zone was not important in BCR [3, 12, 14] whereas other studies found that TZ tumours had significantly longer time to BCR than did PZ/CZ tumours [5, 15–17]. Our study is the first to analyse BCR or early adjustment treatment in TZ and PZ/CZ tumours when margin status and Gleason grade are considered. Our results show that in low-risk tumours TZ vs PZ/CZ behave similarly in margin-positive and margin-negative low-risk cancers. As shown in other studies [5], it appears that a low-risk prostate tumour is a good prognostic cancer regardless of zonal origin. There was also no difference in BCR and time to early treatment between TZ and PZ/CZ high-risk, margin-positive tumours, confirming that high-risk, positive margin tumours have poor prognostics regardless of tumour zone origin [14, 17]. However, this does not seem to be the case for high-risk, margin-negative tumours, where there was a statistically significant reduction in BCR/early treatment for TZ tumours in this cohort compared to PZ/CZ tumours. Differences in tumour egress from the prostate are well described and most PZ/CZ cancers escape the gland along posterolateral penetrating perineural spaces. These are often associated with skip areas which compromises margin assessment. In the BN no such neural spread is present and margin assessment is, therefore, more reliable. This may explain the differences between PSA failure/early therapy in TZ vs PZ or CZ tumours. At the time of RP, the margin negative but high-risk PZ/CZ tumours may have micrometastasis and perineural skip lesions that were not histologically identified.

One limitation of this study is the low rate of pelvic lymph node dissections as 31.7% of all men who underwent RP in WA had a pelvic lymph node dissection. However, there was no statistical difference in the rate of PSA failure or early treatment, in the patients with or without lymph node dissection who had the high-risk, margin-negative, TZ cancer.

## Conclusion

The implication of this analysis is that long-term biochemical cure of prostate cancer is certainly more likely in high-risk TZ cancer than in high-risk PZ or CZ cancer if an isolated positive BN margin is avoided. The clinical importance of such a finding is probably based on the difficulty in assessment of tumour margins in the periphery of the gland. By contrast, margins at the BN are more easily assessed and hence in the subset of high-risk TZ cancer, ensuring a negative BN margin is critical for PSA free cure. Pre-operative prediction based on MRI and Glutaraldehyde-based TZ morphology can surely assist with operation planning and achieving tumour-negative margins.

## Data Availability

Not applicable in this study.
